# Development, Evaluation, and Implementation of a Pan-African Cancer Research Network: Men of African Descent and Carcinoma of the Prostate 

**DOI:** 10.1200/JGO.18.00063

**Published:** 2018-09-27

**Authors:** Caroline Andrews, Brian Fortier, Amy Hayward, Ruth Lederman, Lindsay Petersen, Jo McBride, Desiree C. Petersen, Olabode Ajayi, Paidamoyo Kachambwa, Moleboheng Seutloali, Aubrey Shoko, Mamokhosana Mokhosi, Reinhard Hiller, Marcia Adams, Chrissie Ongaco, Elizabeth Pugh, Jane Romm, Tameka Shelford, Frank Chinegwundoh, Ben Adusei, Sunny Mante, Nana Yaa Snyper, Ilir Agalliu, David W. Lounsbury, Thomas Rohan, Alex Orfanos, Yuri Quintana, Judith S. Jacobson, Alfred I. Neugut, Edward Gelmann, Joseph Lachance, Cherif Dial, Thierno Amadou Diallo, Mohamed Jalloh, Serigne Magueye Gueye, Papa Moussa Sène Kane, Halimatou Diop, Anna Julienne Ndiaye, Amina Sow Sall, Ndeye Coumba Toure-Kane, Ezenwa Onyemata, Alash’le Abimiku, Andrew A. Adjei, Richard Biritwum, Richard Gyasi, Mathew Kyei, James E. Mensah, Julian Okine, Vicky Okyne, Isabella Rockson, Evelyn Tay, Yao Tettey, Edward Yeboah, Wenlong C. Chen, Elvira Singh, Michael B. Cook, Christine N. Duffy, Ann Hsing, Cassandra Claire Soo, Pedro Fernandez, Hayley Irusen, Oseremen Aisuodionoe-Shadrach, Abubakar Mustapha Jamda, Peter Oluwole Olabode, Maxwell Madueke Nwegbu, Olalekan Hafees Ajibola, Olushola Jeremiah Ajamu, Yakubu Garba Ambuwa, Akindele Olupelumi Adebiyi, Michael Asuzu, Olufemi Ogunbiyi, Olufemi Popoola, Olayiwola Shittu, Olukemi Amodu, Emeka Odiaka, Ifeoluwa Makinde, Maureen Joffe, Audrey Pentz, Timothy R. Rebbeck

**Affiliations:** **Caroline Andrews**, **Brian Fortier**, **Amy Hayward**, **Ruth Lederman**, and **Timothy R. Rebbeck**; Dana-Farber Cancer Institute; **Alex Orfanos** and **Yuri Quintana**, Beth Israel Deaconess Medical Center; **Timothy R. Rebbeck**, Harvard T.H. Chan School of Public Health, Boston, MA; **Lindsay Petersen**, **Jo McBride**, **Desiree C. Petersen**, **Olabode Ajayi**, **Paidamoyo Kachambwa**, **Moleboheng Seutloali**, **Aubrey Shoko**, **Mamokhosana Mokhosi**, and **Reinhard Hiller**, Centre for Proteomic and Genomic Research; **Pedro Fernandez** and **Hayley Irusen**, Stellenbosch University and Tygerberg Hospital, Cape Town; **Wenlong C. Chen** and **Elvira Singh**, National Cancer Registry, National Health Laboratory Service; **Wenlong C. Chen**, **Elvira Singh**, **Maureen Joffe**, **Audrey Pentz**, and **Cassandra Claire Soo**, University of Witwatersrand, Johannesburg, South Africa; **Marcia Adams**, **Chrissie Ongaco**, **Elizabeth Pugh**, **Jane Romm**, and **Tameka Shelford**, Center for Inherited Disease Research, Baltimore; **Michael B. Cook**, National Cancer Institute, National Institutes of Health, Bethesda, MD; **Frank Chinegwundoh**, Bart’s Health National Health Services Trust, London, United Kingdom; **Ben Adusei**, **Sunny Mante**, and **Nana Yaa Snyper**, 37 Military Hospital; **Andrew A. Adjei**, **Richard Biritwum**, **Richard Gyasi**, **Mathew Kyei**, **James E. Mensah**, **Julian Okine**, **Vicky Okyne**, **Isabella Rockson**, **Evelyn Tay**, **Yao Tettey**, and **Edward Yeboah**, Korle-Bu Teaching Hospital, Accra, Ghana; **Ilir Agalliu**, **David W. Lounsbury**, and **Thomas Rohan**, Albert Einstein College of Medicine, Bronx; **Judith S. Jacobson**, **Alfred I. Neugut**, and **Edward Gelmann**, Columbia University, New York, NY; **Joseph Lachance**, Georgia Institute of Technology, Atlanta, GA; **Cristine N. Duffy** and **Ann Hsing**, Stanford University, Stanford Cancer Institute, Stanford, CA; **Cherif Dial**, **Thierno Amadou Diallo**, **Mohamed Jalloh**, **Serigne Magueye Gueye**, and **Papa Moussa Sène Kane**, Hôpital Général de Grand Yoff, Institute de Formation et de la Recherche en Urologie et de la Santé de la Famillie; **Halimatou Diop**, **Anna Julienne Ndiaye**, **Amina Sow Sall**, and **Ndeye Coumba Toure-Kane**, Hôpital Aristide Le Dantec, Dakar, Senegal; **Ezenwa Onyemata** and **Alash’le Abimiku**, Institute of Human Virology, H3 African Biorepository Initiative; **Oseremen Aisuodionoe-Shadrach**, **Abubakar Mustapha Jamda**, **Peter Oluwole Olabode**, **Maxwell Madueke Nwegbu**, and **Olalekan Hafees Ajibola**, University of Abuja; **Oseremen Aisuodionoe-Shadrach**, **Abubakar Mustapha Jamda**, **Peter Oluwole Olabode**, and **Maxwell Madueke Nwegbu**, University of Abuja Teaching Hospital, Abuja; **Olushola Jeremiah Ajamu** and **Yakubu Garba Ambuwa**, Federal Medical Center, Keffi; **Akindele Olupelumi Adebiyi**, **Michael Asuzu**, **Olufemi Ogunbiyi**, **Olufemi Popoola**, **Olayiwola Shittu**, **Olukemi Amodu**, **Emeka Odiaka**, and **Ifeoluwa Makinde**, University College Hospital, Ibadan, Nigeria.

## Abstract

**Purpose:**

Cancer of the prostate (CaP) is the leading cancer among men in sub-Saharan Africa (SSA). A substantial proportion of these men with CaP are diagnosed at late (usually incurable) stages, yet little is known about the etiology of CaP in SSA.

**Methods:**

We established the Men of African Descent and Carcinoma of the Prostate Network, which includes seven SSA centers partnering with five US centers to study the genetics and epidemiology of CaP in SSA. We developed common data elements and instruments, regulatory infrastructure, and biosample collection, processing, and shipping protocols. We tested this infrastructure by collecting epidemiologic, medical record, and genomic data from a total of 311 patients with CaP and 218 matched controls recruited at the seven SSA centers. We extracted genomic DNA from whole blood, buffy coat, or buccal swabs from 265 participants and shipped it to the Center for Inherited Disease Research (Baltimore, MD) and the Centre for Proteomics and Genomics Research (Cape Town, South Africa), where genotypes were generated using the UK Biobank Axiom Array.

**Results:**

We used common instruments for data collection and entered data into the shared database. Double-entered data from pilot participants showed a 95% to 98% concordance rate, suggesting that data can be collected, entered, and stored with a high degree of accuracy. Genotypes were obtained from 95% of tested DNA samples (100% from blood-derived DNA samples) with high concordance across laboratories.

**Conclusion:**

We provide approaches that can produce high-quality epidemiologic and genomic data in multicenter studies of cancer in SSA.

## INTRODUCTION

In 2015, approximately 460,000 individuals died of cancer in sub-Saharan Africa (SSA).^1^ By 2035, that figure is expected to more than double, partly as a result of increasing life spans (as a result of decreasing infectious disease mortality), lifestyle changes associated with increasing cancer risk (eg, smoking, obesity), and limited capacities for cancer prevention and treatment. The United Nations Sustainable Development Goals, adopted in 2015, set specific targets for reducing global premature mortality associated with noncommunicable diseases, including cancer.^2^

Cancer of the prostate (CaP) is the leading noncutaneous cancer in men worldwide,^3-5^ and worldwide, men of African descent have higher CaP incidence and mortality than men of other races or ethnicities.^6^ In SSA, the number of CaP deaths per year is predicted to increase from 39,000 in 2015 to 76,000 by 2035.^4^ CaP is more frequently diagnosed at a late (usually incurable) stage in SSA than in other parts of the world,^7-10^ and little is known about the roles of exposure or genetic susceptibility loci in its etiology. CaP has the highest heritability of the common cancers.^11,12^ Many genetic susceptibility loci have been identified in men of European and Asian descent; however, these loci have not generally been validated in men of African descent, underscoring the need for these studies in African-descent populations.

To address those gaps in our knowledge, we developed a multicenter Men of African Descent and Carcinoma of the Prostate (MADCaP) Network. This article describes the scientific principles, research methods, and standardized procedures and protocols developed by MADCaP investigators for our CaP research in SSA. These approaches may serve as a model for other sorely needed cancer research studies in SSA.

## METHODS

### Network Organization

**Fig 1:**
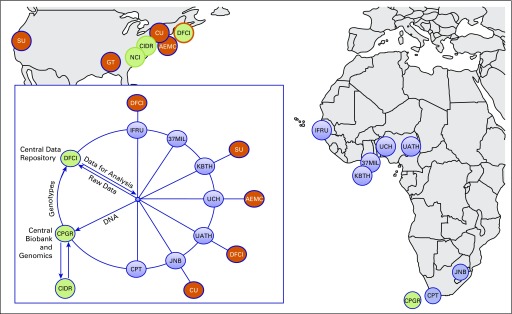
Men of African Descent and Carcinoma of the Prostate (MADCaP) organizational and collaborative structure. African centers (blue circles) include 37 Military Hospital (37MIL), Accra, Ghana; Stellenbosch University and Tygerberg Hospital (CPT), Cape Town, South Africa; Institut de Formation et de la Recherche en Urologie (IFRU), Dakar, Senegal; Wits Health Consortium and National Health Laboratory Service (JNB), Johannesburg, South Africa; Korle-Bu Teaching Hospital (KBTH), Accra, Ghana; University of Abuja Teaching Hospital (UATH), Abuja, Nigeria; and University College Hospital (UCH), Ibadan, Nigeria. US partner institutions (orange circles) include Albert Einstein College of Medicine (AECM), Bronx, New York; Columbia University (CU), New York, New York; Dana-Farber Cancer Institute (DFCI), Boston, Massachusetts; Georgia Institute of Technology (GT), Atlanta, Georgia; and Stanford University (SU), Palo Alto, California. Central resource facilities (green circles) include the Centre for Proteomics and Genomics Research (CPGR), Cape Town, South Africa, and DFCI. Other collaborating MADCaP partners include the Center for Inherited Disease Research (CIDR) and the Intramural Program of the National Cancer Institute (NCI).

Figure 1 depicts the MADCaP Network’s organizational structure, which has seven participant accrual centers in Ghana, Nigeria, Senegal, and South Africa with several SSA and US centers partnered to enhance capacity building and offer mentorship. Additional research partners include the National Cancer Institute (Bethesda, MD), the Center for Inherited Disease Research (CIDR; Baltimore, MD), the Centre for Proteomics and Genomics Research (CPGR; Cape Town, South Africa), and the Georgia Institute of Technology (Atlanta, GA).

### Study Design

In this clinic-based case-control study, eligible participants were men older than age 30 years residing in each MADCaP center’s catchment area and self-identifying as black African with no known or self-reported European, Middle Eastern, or Asian ancestry. All participants were required to reside within a prespecified catchment area surrounding the clinical ascertainment sites. Information about town and country of birth and residential location for the past 10 years was queried. As with patients, the lower age limit for controls was 30 years. The lower bound for inclusion was set at age 30 years on the basis of the earliest age of a CaP diagnosis recorded in any of our centers, which occurred before age 40 years. Eligible patients with CaP, ascertained in urology and oncology clinics or through primary referral, must have had a histologically confirmed first primary CaP of any stage, grade, or pathologic classification. Patient status was confirmed by pathologic diagnosis and medical record review using a standardized abstraction form. Only incident patients, whose first diagnosis of CaP occurred no more than 6 months before study contact, were eligible. Men previously diagnosed with cancer at any other center were excluded.

Controls were frequency matched to patient cases by age, ascertained through nonurology and nononcology clinics (including orthopedics, internal medicine, family medicine, general surgery, GI, geriatrics, neurosurgery, dermatology, cardiology, and ophthalmology clinics) at participating SSA institutions (Fig 1) residing within the catchment area from which the patients with CaP were drawn, and had no history of cancer. The controls represent a wide range of other diagnoses or no diagnosis and may have received examinations including cancer screening, particularly digital rectal exams or prostate-specific antigen tests. They may have been diagnosed with cancer subsequently but did not arrive at the clinics specifically for cancer diagnosis, treatment, or screening. Ascertainment of all participants was undertaken without regard to family history of cancer or any other traits.

### Regulatory Requirements, Ethical Considerations, and Oversight

To comply with local, national, and international regulations governing human subjects research and data sharing, we implemented a series of regulatory protocols. Center and study staff certifications were obtained including US-required federal-wide assurances,^13^ System for Award Management registration (including Data Universal Numbering System number and North Atlantic Treaty Organization Commercial and Government Entity code),^14^ Electronic Research Administration Commons registration, federal conflict of interest statements, and Collaborative Institutional Training Initiative human subjects research training.^15^ Local (sometimes national) and centralized institutional review boards approve study protocols annually, following local laws, regulations, and guidelines, and study compliance is monitored frequently following the Office for Human Research Protections international compilation of human research standards.^16^ US Department of State compliance documents were obtained and distributed to ensure proper implementation of current policies and embargoes.^17^

All participating institutions signed data use agreements and material transfer agreements. Import and export permits were generated enabling biosamples to be shipped to the CPGR in Cape Town, South Africa, where all central processing, biobanking, and genotype screening are conducted. In addition, MADCaP investigators formed a series of oversight working groups.

Each of our centers and the overall study obtained approval for the ethical conduct of the research. These approvals are in accordance with both local and international principles. We took a series of steps to ensure the welfare of our research participants. First, the potential harms to the research participant were minimal. These included minor bruising at the phlebotomy site during peripheral-blood collection. The remaining data collection issues were also minimal risk because they involved questionnaires and medical record abstraction. Second, patient information was protected using standards similar to those outlined in the Health Insurance Portability and Accountability Act. Although the Health Insurance Portability and Accountability Act is a US regulation not in effect in African countries, we have based our patient confidentiality and privacy standards on those regulations, accounting for local laws and regulations that may differ by country. Finally, although the risks to the participants were minimal, the individual benefits to the participants were also small; we did not provide the results of our research to the individual participant. All data are presented in aggregate only. However, the potential benefits to African men may be large as we understand and translate our research results to the clinical and public health of the populations being studied.

### Biosample, Biobanking, and Laboratory Resources

We standardized biosample collection processes to facilitate consumable and reagent sourcing and obtained high-quality DNA selecting Qiagen’s (Hilden, Germany) QIAamp DNA Blood Midi or Mini Kits on the basis of availability, cost effectiveness, DNA quality, and yield. We collected at least 7 mL of blood in EDTA vacutainer tubes from each participant, maintaining them at 4°C while in transit to the local laboratory and subsequently storing them at −30°C before DNA extraction. When a participant did not provide a blood sample, centers could collect a saliva sample using the Oragene OG-500 kit (DNA Genotek, Kanata, Ontario, Canada).

CPGR staff visited each SSA center providing training videos supporting biosample protocol adoption and local capacity building.^18^ Common quality control (QC) processes have been implemented to ensure that defined QC metrics are obtained. The QC parameters for DNA include A_260/_A_280_ ratios between 1.7 and 2.1; A_260/_A_230_ ratios ≥ 1.5; and visualization of high-molecular-weight DNA after gel electrophoresis.

DNA was shipped from each SSA center to CPGR at ambient temperature in DNAstable 2D Barcode 96-well Tube Plates (Matrix; Biomatrica, San Diego, CA) and stored upon receipt at the CPGR at ambient temperature until processed. After sample resuspension and removal of aliquots for QC purposes, DNA was stored at −80°C for long-term storage before downstream processing.

None of our centers had serious power supply issues. However, each of our study centers had generator backups and power surge protectors for the freezers in which our samples were stored. Backup generators kicked in within seconds of a power failure. In addition, we stored our DNA samples in DNA-stable plates, which maximizes DNA quality during shipping as well as potentially variable storage conditions.

### Data Elements, Collection, and Management

After multiple teleconferences and an in-person meeting (January 2017), we finalized common data elements and study protocols. We created two Web-accessible, password-protected databases, residing on secure servers—a Research Electronic Data Capture database^19^ to provide identifiable tracking of participant recruitment, and a DatStat Illume database (DatStat, Seattle, WA) for deidentified storage of study data that include no personal identifying information other than the patient’s date of birth. Each study participant was assigned a unique study identification number, concatenated with center identification, year of accrual, and patient case or control status. Center staff received remote database training^20^ via training guides and tutorials. Videos were available for ongoing training and support.^18^ The Data Coordinating Center (DCC) monitored data collection, ensuring there were no duplicate identifiers, and generated missing data reports.

**Fig 2:**
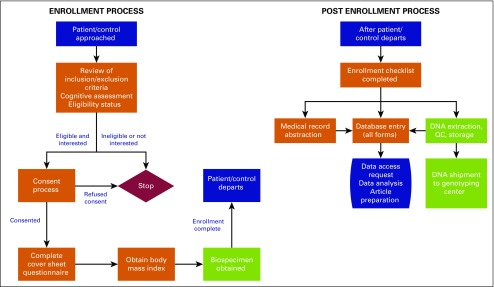
Men of African Descent and Carcinoma of the Prostate study schema separated into the enrollment and postenrollment processes. QC, quality control.

A synopsis of the data collection processes is presented in Figure 2. Staff at each SSA center collected data via paper surveys, entering the data using the unique participant identifier. Survey modules relied on authentication, by means of login credentials and encryption, to maintain security. Secure https connections were used with 128-bit encryption and signed Secure Sockets Layer certificates to enable the highest level of security. Data could be entered live or, because some centers have difficulty accessing the Internet as a result of low bandwidth, statically using DatStat’s Remote Data Collection with bulk uploading. Center investigators kept original data at their local centers. Web-based survey data were stored on secure, password-protected servers at DatStat’s headquarters in Seattle, Washington. For QC, 10% of all data were re-entered by the DCC.

### Pilot Studies

We conducted two pilot studies to evaluate processes and protocols.

#### Data collection.

All SSA study staff were trained in person. Data collection was monitored to evaluate protocol adherence and the capacity of each center to collect and enter high-quality data.

#### Biosampling and genotyping.

We evaluated each center’s ability to collect, process, and ship DNA across Africa and to use the DNAstable plate for stabilization, storage, and shipment of DNA at room temperature to the CIDR and CPGR. Center staff processed both prospectively and retrospectively collected samples (biosamples previously obtained after unstandardized protocols). By studying these retrospectively collected samples, we could evaluate samples collected under a wide variety of conditions in SSA. Centers were encouraged to provide a range of samples with optimal and suboptimal QC metrics, including low A_260_/A_230_ ratios and partially degraded samples, submitting 1 to 3 µg of DNA in DNAstable plates after the drying down of DNA samples in a laminar flow hood. The 265 biosamples collected in this pilot were evaluated in parallel experiments using the UK Biobank Axiom Array (Affymetrix, Santa Clara, CA) at CPGR. CIDR tested 234 DNA samples, assessing QC by running e-gels, Picogreen, and/or Nanodrop and the Illumina QC Array (Illumina, San Diego, CA). CPGR tested 223 samples using TaqMan OpenArray Genotyping Barcodes (Thermo Fisher Scientific, Waltham, MA). By design, some samples were sent to both CIDR and CPGR to evaluate reproducibility of results. Duplicate samples were included to help determine technical reproducibility within and across genotyping centers and assess the overall minimal sample quality threshold required.

## RESULTS

### Standardized Procedures and Protocols

We developed a study binder consisting of 11 protocols and 15 data collection forms to provide comprehensive guidance to the SSA centers relating to regulatory, ethical, and data collection procedures. The data collected for this study include basic demographic and clinical characteristics, epidemiologic risk factors, and pathology features.

### Communications and Training

We created a communication platform (www.madcapnetwork.org)^21^ built on the Alicanto social learning platform (www.alicantocloud.com) supporting creation of public and private groups, with document sharing, threaded discussion, and videoconferencing using Zoom software (https://zoom.us). We used these communication tools initially to ensure standardization across study centers and continue to use them to facilitate and monitor study progress, share information, and nurture collaboration. In its first year, the Web site had 1,075 unique visitors, 1,875 visitors’ sessions, 29,030 page views, 112 registered users, and 11 groups available to registered users. The MADCaP study enrollment video^22^ guides the project manager through all enrollment steps. The Web site was recently awarded a Gold Davey award in the Education category by the Academy of Interactive and Visual Arts. 

### Data Collection Pilot

Compliance documentation including institutional review board protocol approvals, document translation (French, IsiXhosa, and Afrikaans), and material transfer and data use agreements was obtained. Across seven centers, a total of 186 patients and 122 controls were enrolled onto the pilot study (Table 1). To monitor data integrity, two-pass verification (double data entry) was undertaken on 275 (10%) different pilot study survey sets to verify center entry accuracy. A high concordance of 95% to 98% was achieved between participating centers and verification data entry (Table 2). Double data entry will continue using a 10% subset of the total sample as in the pilot throughout the study’s duration. Additional quality assurance includes ongoing data cleaning by the DCC to identify incomplete, incorrect, or inaccurate data; the DCC circulates monthly reports describing erroneous data points and specific errors to participating centers to ensure error correction.

**Table 1:**
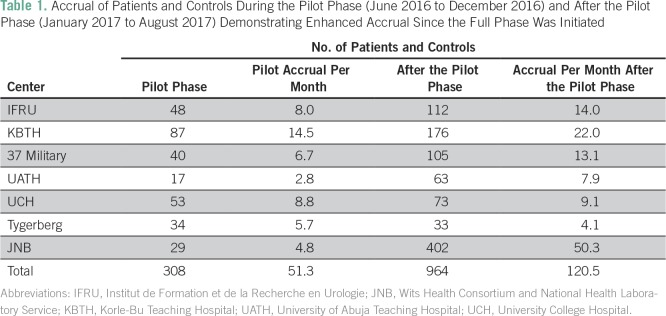
Accrual of Patients and Controls During the Pilot Phase (June 2016 to December 2016) and After the Pilot Phase (January 2017 to August 2017) Demonstrating Enhanced Accrual Since the Full Phase Was Initiated

**Table 2:**
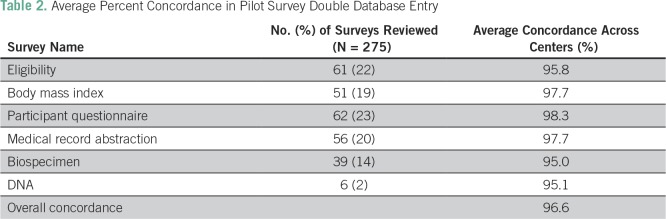
Average Percent Concordance in Pilot Survey Double Database Entry

### Biosampling and Genotyping Pilot

For the laboratory pilot, documentation was generated, and the SSA centers successfully transported both retrospectively and prospectively collected biosamples to the CPGR and CIDR. After DNA extraction, centers performed QC analysis and shipped an aliquot of each sample to the CPGR for corroborative QC analysis. Both CIDR and CPGR performed QC analysis including Nanodrop, gel electrophoresis, and/or Picogreen assays to measure the quality, quantity, and integrity of the biosamples. Appendix Table A1 shows the high correlation in QC analysis between SSA centers and the CPGR, with the correlation in median DNA yield being particularly high (Spearman correlation coefficient = 0.964; *P* < .001).

Average center call rate from the Illumina Infinium QC array was > 99% for blood and buffy coat samples, whereas samples derived from buccal swab had lower call rates (≤ 92%). Similar performance of samples was observed for the OpenArray Genotyping Barcode Panel for 60 single nucleotide polymorphisms. The samples with the lowest success in both pretesting assays were derived from buccal swabs, which showed significant DNA degradation and displayed the poorest QC metrics (Table 3) and lowest call rates. For all centers, intact high-molecular-weight DNA was obtained in sufficient quantities, but DNA degradation was more common in the buccal swab samples compared with blood or buffy coat.

**Table 3:**
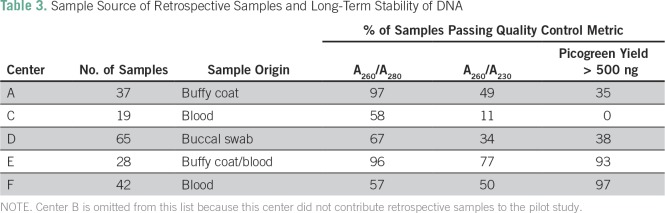
Sample Source of Retrospective Samples and Long-Term Stability of DNA

**Fig 3:**
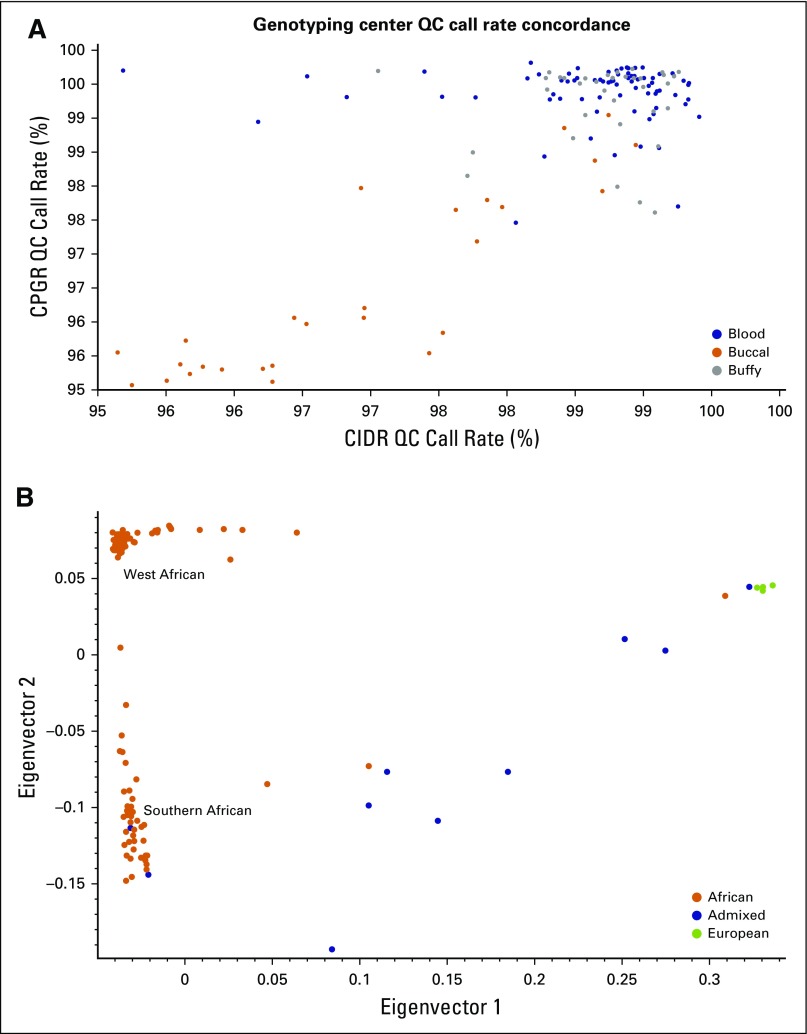
Results of genotype analyses of study samples. (A) Success rate of samples assayed on the Axiom Array. (B) Results of principal components analysis to evaluate genomic ancestral population groups where different colors represent different population groups; orange represents African, blue represents admixed, and green represents European. Eigenvalues indicates the amount of variation in the sample data. Eigenvectors are the linear combinations showing how variables contribute to each axis. CIDR, Center for Inherited Disease Research; CPGR, Centre for Proteomics and Genomics Research; QC, quality control.

Of the samples assayed on the UK Biobank Axiom Array at both CIDR and CPGR, approximately 47% were derived from blood, 23% from buffy coats, and 30% from buccal swabs representing various DNA samples. Samples were included from three different African countries (Senegal, Ghana, and South Africa). Fig 3A shows the representative success rate for these sample types, with strong concordance between the two genotyping centers. Like the pretesting assays, samples derived from blood and buccal swabs performed optimally, displaying high call rates (ie, > 97%), whereas only buccal swab–derived samples with intact high-molecular-weight DNA passed QC. In addition, genomic ancestry of the genotyped individuals was evaluated (Fig 3B), with the majority of participants being genomically of African ancestry, whereas a few South African individuals (n = 6) were confirmed to have European or admixed ancestry. This analysis allows for comparison with self-identified population groups and will be used to confirm the ancestry of all study participants in future genome-wide association studies.

## DISCUSSION

SSA has been greatly under-represented in studies of cancer.^23^ However, we have now developed a multicenter research network to study CaP in SSA men. The results presented provide strong evidence that each center can ascertain controls and patients and obtain the required data to achieve the study aims, highlighting the importance of the pilot phase in establishing appropriate and adequate study implementation. The MADCaP Network serves as a paradigm for multicenter cancer research in SSA and among researchers and clinicians in SSA centers with US partners. This collaboration is building capacity and sustainability for cancer research in SSA. SSA centers maintain control of their own studies and may publish independently. Contributing SSA principal investigators (PIs) may also use consortium resources and data to address questions of interest. Data sharing with center PI permission and through confidentiality and data use agreements protects investigators and allows free exchange of data and ideas among consortium members.

This report describes how SSA centers can become equipped to perform participant ascertainment, data and biosample collection, and DNA processing. QC analysis of prospectively collected data and biospecimens indicated that each center could adequately follow the project’s protocols and procedures to generate data and to extract, store, and ship high-quality biosamples. The biosampling pilot study confirmed the suitability of Biomatrica plates for shipping DNA samples from each African center to the CPGR and CIDR at ambient temperature, validating its continued use for shipping samples for the main study and thus eliminating the need for dry ice shipments, an important shipping option in Africa. Moreover, DNAstable, the DNA stabilizing reagent used to coat the plate wells, did not negatively affect the DNA integrity or sample performance on the Axiom assays using the UK Biobank Array.

A high percentage of retrospectively collected buccal samples failed the assay as a result of sample degradation. Blood and buffy coat samples, irrespective of sample age, performed well, with DNA of high molecular weight and little or no degradation. Older samples that had gone through repeat freeze-thaw cycles provided a lower DNA yield, although they still contained sufficient DNA to perform required assays. The genotyping assays used can also tolerate lower than expected QC metrics, in particular with respect to the A_260_/A_230_ ratio. These results indicate that prospective and retrospective samples will produce a high call rate for genotypes using genotyping assays on a variety of platforms. Moving forward, a standard operating procedure for buccal cell collection, preprocessing, storage, and transport will be optimized and universally used for DNA for MADCaP.

We have identified principles critical to development of a sustainable research network that has clinical and public health impact. Elements required for successful network development include the commitment of a dedicated, well-connected, and influential local PI willing to promote and mentor junior researchers. An academic environment enabling research is also required, particularly to provide dedicated research time, given the clinic loads of most clinician investigators. Appropriate training and academic career ladders may need to be developed or tailored to permit committed individuals to dedicate effort to a research career. Some SSA institutions do not yet provide these pathways.^24,25^ Successful networks may need to identify incentives, rewards, and recognition for research faculty and staff, modeling a locally appropriate change management approach, and develop easy-to-use platforms that can scale to larger size studies as well as studies of other noncommunicable diseases. The research itself must be developed in terms of achievable goals with realistic timelines. Finally, the network must provide clear expectations and metrics for collaborators’ contributions and communication, including regular calls and bidirectional center visits. We have also used these lessons to identify key overarching goals for the network, including the development of improved research infrastructure considering the needs and setting in SSA.

We also identified several challenges in developing our network. We initially had trouble identifying well-trained, experienced technical staff, but we found we could identify and train center staff to meet our research needs. We observed the value of regular, consistent, targeted communication to address the needs of individual centers and keep the project on track. Early in network development, we learned that we needed to identify realistic study goals and timelines to avoid unmet expectations. We had to set common standards and make sure that protocols were consistent with the centers’ previous experiences and expectations. The development of tools for protocol and data harmonization by a consensus helped avoid unnecessary conflict among groups with different past research experiences. Our adoption of Web-based tools and a communications platform, through which each investigator and staff member could readily find documentation on the study protocols and progress, helped avoid confusion or miscommunication. A major challenge for data collection was being able to obtain consistent Internet access. Once this limitation was identified, we switched from a system of online data entry to a protocol allowing local data entry and bulk data upload at centers having difficulty accessing the Internet. On the basis of our experience to date, we have realistic expectations of returns on the substantial investments of money, resources, and time that MADCaP entails, including improved research infrastructure, a trained local workforce, improved research capacity, and potentially important contributions to science.

## Figures and Tables

**Table A1:**
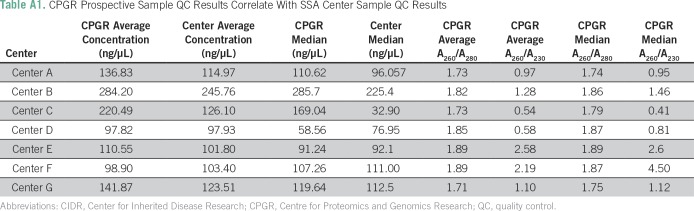
CPGR Prospective Sample QC Results Correlate With SSA Center Sample QC Results
